# Impact of vertical integration on patients’ use of hospital services in England: an analysis of activity data

**DOI:** 10.3399/BJGPO.2023.0231

**Published:** 2024-05-29

**Authors:** Catherine Saunders, Charlotte Davies, Manbinder Sidhu, Jon Sussex

**Affiliations:** 1 Department of Psychiatry, University of Cambridge, Cambridge, UK; 2 RAND Europe Community Interest Company, Cambridge, UK; 3 University of Birmingham, Health Services Management Centre, Birmingham, UK

**Keywords:** social sciences, statistics, hospital referrals, general practice

## Abstract

**Background:**

Debate surrounding the organisation and sustainability of primary care in England highlights the desirability of a more integrated approach to patient care across all settings. One such approach is ‘vertical integration’, where a provider of specialist care, such as a hospital, also runs general practices.

**Aim:**

To quantify the impact of vertical integration on hospital use in England.

**Design & setting:**

Analysis of activity data for NHS hospitals in England between April 2013 and February 2020.

**Method:**

Analysis of NHS England data on hospital activity, which looked at the following seven outcome measures: accident and emergency (A&E) department attendances; outpatient attendances; total inpatient admissions; inpatient admissions for ambulatory care sensitive conditions; emergency admissions; emergency readmissions; and length of stay. Rates of hospital use by patients of vertically integrated practices and controls were compared, before and after the former were vertically integrated.

**Results:**

In the 2 years after a GP practice changes, for the population registered at that practice, compared with controls, vertical integration is associated with modest reductions in rates of A&E attendances (2% reduction [incidence rate ratio {IRR} 0.98, 95% confidence interval {CI} = 0.96 to 0.99, *P*<0.0001]), outpatient attendances (1% reduction [IRR 0.99, 95% CI = 0.99 to 1.00, *P* = 0.0061]), emergency inpatient admissions (3% reduction [IRR 0.97, 95% CI = 0.95 to 0.99, *P* = 0.0062]), and emergency readmissions within 30 days (5% reduction [IRR 0.95, 95% CI = 0.91 to 1.00, *P* = 0.039]), with no impact on length of stay, overall inpatient admissions, or inpatient admissions for ambulatory care sensitive conditions.

**Conclusion:**

Vertical integration is associated with modest reductions in use of some hospital services and no change in others.

## How this fits in

A small but growing number of general practices in the NHS in England are run by trusts (NHS organisations providing specialist health care). There is little evidence whether and to what extent this type of vertical integration between primary and secondary care affects patients’ use of hospital services. This paper reports the first national-level statistical analysis to address that question.

## Introduction

At the founding of the NHS in the UK in 1948, general practices, providers of primary medical care, were kept organisationally separate from hospitals and other providers of secondary care. This arrangement lasted for decades but since 2015, some general practices have started to be run by secondary care provider organisations, which are known as ‘trusts’ in the NHS in England. This is a form of vertical integration of health care. National policy for the NHS in England has concentrated on horizontal integration between general practices.^1–4^ Vertical integration has emerged in response to local initiative rather than national mandate. Davies *et al* identified in England at the end of March 2021 a total of 85 general practices (operating from 116 sites) being run by 26 trusts.^5^ These represent one in 80 general practices and one in nine trusts.

The most important driver for trusts to run general practices has been found to be to safeguard continued delivery of care local to where patients live and thereby enable better management of how patients use hospital services.^6,7^ Associations between better access to primary care and lower rates of hospital A&E attendance, and between better continuity of GP consulted and lower rates of elective and emergency hospital admissions, have been found in other studies.^8–11^ Vertical integration creates opportunities for developing patient services in primary care settings and better integrating them with secondary care to achieve improved patient outcomes and patient experiences of health care.^6,7^ Sustaining primary care, alongside growing patient demand and workforce constraints, is a matter of increasing concern.^12^ Better integration and coordination of primary and secondary care services are among suggestions to address it.^1^ Such arrangements have been tried internationally.^13–15^ A study of vertical integration between an acute hospital and 10 general practices in the West Midlands of England, found that it was associated with a reduced rate of unplanned hospital admissions and readmissions, and corresponding opportunities for reductions in hospital costs.^16^ In 2022, the Secretary of State for Health and Social Care in England stated interest in encouraging vertical integration.^17,18^

We report here a study across all instances of vertical integration in the NHS in England up to 31 March 2021 to determine the impact of vertical integration on patients’ use of hospital services. This work was part of a larger, mixed-methods project to evaluate the impact of vertical integration on efficiency outcomes and patient experience; a detailed report of which is published elsewhere.^19^

## Method

We compared practices that underwent vertical integration with trusts with control practices in England using data from April 2013–February 2020 on patients’ use of secondary care.

### Outcomes

We used person-level Hospital Episode Statistics (HES) activity data from NHS Digital (which has now merged with NHS England),^20^ accessed under a Data Sharing Agreement, to evaluate the impact of vertical integration for seven outcome measures of secondary care utilisation, as shown in Table 1.

**Table 1.** Secondary care utilisation measures

Outcome	Details
A&E attendances	All A&E attendances at all types of emergency care departments and providers were included. For financial year 2019–2020 we used HES A&E data rather than the new Emergency Care Data Set, to maintain consistency across the whole analysis timeframe. Multiple attendances on the same day were only included once.
Outpatient attendances	Only outpatient appointments marked as having been attended were included.
Inpatient admissions	Inpatient admissions are recorded in HES as a series of ‘Finished Consultant Episodes’ (time spent under a particular consultant’s care). Sometimes a patient’s stay in hospital includes successive periods under the care of different consultants. We linked these episodes to form single admissions using the University of York Centre for Health Economics 'Continuous Inpatient Spell' definition. Because the person identifier for HES changed during the 2019–2020 financial year, we used the mapping files provided by NHS Digital to allow for continuous inpatient spells that started in financial year 2018–2019 but finished in 2019–2020 (the date across files where the person identifier changed).
Inpatient admissions with an ambulatory care sensitive condition	Admissions were flagged if they were related to an ambulatory care sensitive condition (ACSC) based on the classification used by Bardsley *et al*.^24^
Emergency inpatient admissions	Admissions were defined as emergency admissions based on the HES data classification.
Emergency readmissions	We used the University of York Centre for Health Economics definition for readmissions within 30 days of discharge, and included only emergency-coded admissions.
Length of stay	Days calculated for continuous inpatient spells, and included based on the date of admission.

A&E = accident and emergency. HES = Hospital Episode Statistics

### Intervention

We used (primarily) trust statutory financial reporting, but combined with searches of primary care workforce data, Care Quality Commission (CQC) practice ownership datasets, and reviews of awarded contracts for delivering primary care services, to identify where vertical integration had taken place.^19^ We identified the date of vertical integration primarily from trusts’ statutory financial reports, but also from practice websites and local and trade press. All vertically integrated practices are identified using a unique practice code, which is an organisation code that uniquely identifies each general practice in England.

### Counterfactual

Vertical integration is one of several new organisational models affecting NHS primary care. Horizontal merger between general practices is a second; and use of Alternative Provision of Medical Services (APMS) contracts is a third. As a counterfactual, we used patients from a random sample of ‘stable’ practices; by which we mean practices that are neither vertically integrated, nor merged with other practices, nor changed contract type since April 2013 but remained on a General Medical Services (GMS) contract. As a supplementary analysis, we compared the impact of vertical integration for practices integrated with acute hospital trusts compared with those integrated with community trusts (we use the term ‘community trust’ as shorthand for any trust that does not run an acute hospital). This approach enabled us to match on the challenges that practices may experience before vertically integrating and explore the impact of integrating specifically with acute hospitals. Details of the methodological work underpinning this counterfactual approach overall are published separately.^19^

To identify practices that change contract type, we used annually reported data from NHS Digital.^21^ To identify horizontal mergers between practices we used person-level HES data from April 2013–February 2020, tracing the new practice codes of patients attending hospital outpatient services where earlier practice codes subsequently disappeared.^19,22^

### Analysis

We obtained data from NHS Digital covering the period from 1 April 2013–1 February 2020 because of the profound impact of the COVID-19 pandemic on secondary care utilisation after February 2020. To balance length of follow-up period against the number of vertically integrated practices in the resulting sample, we included practices for which follow-up of at least 2 years pre-integration and 2 years post-integration was possible. Thus, these are practices where vertical integration occurred between 1 April 2015 and 1 February 2018. Where vertically integrated practices had undergone horizontal mergers during the analysis period (1 April 2013–1 February 2020), we included the practice as a single merged practice in the analysis across the whole period.

In our first analysis, we described secondary care utilisation in the pre-intervention period before any practice underwent vertical integration (2014–2015) for all vertically integrated and control practices.

In our second analysis, we used a multivariable difference in difference framework adjusting for person-level age, sex, deprivation, calendar month, and year. We used a separate categorical flag for each month to account for both secular trends and seasonal variation, a flag for whether practices were intervention (vertical integration) or control practices, and a flag (in intervention practices) for whether the time period included was before or after the date of the intervention. We adjusted separately for time (as a linear variable) before and after the date of the integration in intervention practices to account and control for potential differences in pre-intervention trends in intervention practices compared with control practices. We additionally included the following: a random effect for practice; a random slope for the intervention to allow the impact of the intervention to vary between practices; and a random slope for year to allow trends over time to vary between practices. For intervention practices, we only included the 2 years before and after the intervention, but for control practices, we included all time periods to allow adjustment for secular trends. For all outcomes except length of stay, we used Poisson models, for length of stay we used a linear model.

In a further multivariable difference in difference analysis, only vertical integration practices are included and practices that integrated with an acute hospital trust are compared directly with practices that integrated with a community trust.

## Results

Fifty-nine practices vertically integrated between 1 April 2015 and 1 February 2018, and horizontal mergers by February 2020 mean that these are included in the analysis as 42 distinct practices across the whole study period. Three of these practices have been excluded because of data-quality issues, particularly around organisation coding in HES identified in preliminary analyses. Thus, 39 vertically integrating practices are included in the analysis; 25 integrated with acute trusts and 14 with community trusts. Five hundred control practices were sampled, with 492 included after exclusions for poor data quality.

Table 2 shows the median rates at which patients of practices that went on to vertically integrate were using hospital services in financial year 2014–2015. There were 0.31 (median) A&E attendances per patient per year at vertically integrating practices, slightly higher than the 0.29 A&E attendances per patient in control practices. Vertically integrating practices also had slightly higher median rates of inpatient admissions and readmissions than control practices. The median rate of outpatient attendances was the same in vertically integrating and control practices. The mean hospital length of stay for patients at the median vertically integrating practice was slightly shorter, at 2.84 days, than at the median stable practice, 2.94 days. Median outpatient attendance rates were lower and emergency readmission rates higher in practices that integrated with community compared with acute trusts.

**Table 2.** Pre-intervention (2014–2015) rates of use of hospital services by patients of vertical integration practices and a sample of stable practices

	Vertical integration practices (all) (***n* = 39**)	Vertical integration: acute trusts (***n*= 25**)	Vertical integration: community trusts (***n* = 14**)	Control practices (***n* = 492**)
**Secondary care use; rate per person per year; median (IQR) across practices in each group**				
A&E attendances	0.31 (0.26–0.39)	0.30 (0.25–0.39)	0.33 (0.29–0.40)	0.29 (0.24–0.36)
Outpatient attendances	1.27 (1.15–1.59)	1.32 (1.15–1.59)	1.24 (1.15–1.58)	1.27 (1.12–1.47)
Inpatient admissions	0.29 (0.26–0.31)	0.29 (0.28–0.32)	0.29 (0.25–0.31)	0.26 (0.22–0.29)
Inpatient admissions for ACSC	0.03 (0.02–0.04)	0.03 (0.02–0.04)	0.03 (0.02–0.04)	0.02 (0.02–0.03)
Emergency inpatient admissions	0.09 (0.08–0.11)	0.09 (0.09–0.12)	0.10 (0.08–0.11)	0.08 (0.07–0.10)
Emergency readmissions (per 100 people per year)	1.82 (1.50–2.63)	1.78 (1.69–2.41)	1.96 (1.39–2.76)	1.70 (1.33–2.07)
**Length of stay; mean days per admission; median (IQR) across practices in each group**				
Length of stay	2.84 (2.62–3.21)	2.84 (2.69–3.21)	2.78 (2.46–3.39)	2.94 (2.57–3.35)

ACSC = ambulatory care sensitive condition. A&E = accident and emergency. IQR = interquartile range.

The impact of general practices vertically integrating with trusts on the rates at which their patients use hospital services are shown in Table 3. On average, in the 2 years after becoming vertically integrated, the rate of A&E attendances in the integrating practices fell by (a modest, but statistically significant) 2% (incidence rate ratio [IRR] 0.98 [95% confidence interval CI = 0.96 to 0.99], *P*<0.0001). Vertical integration is also associated with a 1% reduction in the rate of outpatient attendances (IRR 0.99 [95% CI = 0.99 to 1.00], *P*=0.0061); a 3% reduction in the rate of emergency inpatient admissions (IRR 0.97 [95% CI = 0.95 to 0.99], *P* = 0.0062); and a 5% reduction in the rate of emergency readmissions (IRR 0.95 [95% CI = 0.91 to 1.00], *P* = 0.039) when it is first introduced. There was no evidence that vertical integration had any impact on length of inpatient stay. The falls in A&E and outpatient attendance rates appear to be temporary, as these grow at faster rates than control practices during the 2 years of follow-up after vertical integration.

**Table 3.** The impact on hospital use of vertical integration compared with control practices

	Step change at time of intervention compared with 'stable' GMS control practices		Additional yearly change after the intervention	
**Outcome**	**IRR (95% CI**)	***P*-value**	**IRR (95% CI**)	***P*-value**
A&E attendances	0.98 (0.96 to 0.99)	<0.0001	1.02 (1.00 to 1.04)	0.012
Outpatient attendances	0.99 (0.99 to 1.00)	0.0061	1.02 (1.00 to 1.03)	0.0064
Inpatient admissions	1.00 (0.99 to 1.01)	0.94	0.97 (0.96 to 0.99)	0.0002
Inpatient admissions for ACSC	0.98 (0.94 to 1.01)	0.23	0.98 (0.96 to 1.01)	0.24
Emergency inpatient admissions	0.97 (0.95 to 0.99)	0.0062	1.00 (0.98 to 1.01)	0.61
Emergency readmissions	0.95 (0.91 to 1.00)	0.039	1.01 (0.97 to 1.04)	0.74
	**Difference (95% CI**)	***P*-value**	**Difference (95% CI**)	***P*-value**
Length of stay (days)	0.09 (−0.11 to 0.28)	0.51	–0.03 (–0.11 to 0.05)	0.51

ACSC = ambulatory care sensitive condition. A&E = accident and emergency. GMS = General Medical Services. IRR = incidence rate ratio.

Comparing practices that vertically integrate with acute hospital trusts with practices that integrate with community trusts (see Table 4), the rate of A&E attendances shows a differential step-change fall at the date of vertical integration that is 3% more when integration is with an acute hospital trust than when it is with a community trust (IRR 0.97 [95% CI = 0.95 to 0.99], *P* = 0.01). But the rate of inpatient admissions is 4% more in vertical integration with acute trusts than with community trusts (IRR 1.04 [95% CI =1.01 to 1.06], *P* = 0.0095). There are no other statistically significant differences in step changes between vertical integration with an acute trust or a community trust.

**Table 4.** The impact on hospital use of vertical integration with acute hospital trusts compared with vertical integration with community trusts

	Percentage point step change for acute minus that for community vertical integration practices		Additional yearly change after the intervention for acute compared with community vertical integration	
	**IRR (95% CI**)	***P*-value**	**IRR (95% CI**)	***P*-value**
**IRR per patient per year**				
A&E attendances	0.97 (0.95 to 0.99)	0.010	1.02 (0.98 to 1.06)	0.32
Outpatient attendances	1.00 (0.99 to 1.01)	0.72	1.13 (1.03 to 1.24)	0.013
Inpatient admissions	1.04 (1.01 to 1.06)	0.0095	1.15 (0.92 to 1.45)	0.22
Inpatient admissions (ACSC)	1.02 (0.95 to 1.10)	0.64	0.95 (0.70 to 1.29)	0.75
Emergency inpatient admissions	1.02 (0.98 to 1.06)	0.32	0.82 (0.65 to 1.04)	0.10
Emergency readmissions	1.02 (0.95 to 1.11)	0.56	1.09 (0.86 to 1.37)	0.49
	**Difference (95% CI**)	***P*-value**	**Difference (95% CI**)	***P*-value**
**Days**				
Length of stay	–0.08 (–0.23 to 0.06)	0.26	–0.08 (–0.23 to 0.07)	0.31

ACSC = ambulatory care sensitive condition. A&E = accident and emergency. IRR = incidence rate ratio

Although we found no differential impact between the two types of trusts at the date of vertical integration with respect to outpatient attendances, we note that in practices that vertically integrate with acute hospitals, the post-integration rate of outpatient attendances does increase over the next 2 years at a considerably faster rate than in practices that integrate with community trusts (IRR 1.13 [95% CI = 1.03 to 1.24)] per year, *P* = 0.013). There are no statistically significant differences in post-integration trends in rates of other types of hospital use, comparing practices that integrate with acute trusts with practices that integrate with community trusts (Table 4).

## Discussion

### Summary

Our work has provided evidence of the impact on hospital utilisation of vertical integration in the NHS in England between trusts (providers of secondary care) and general practices (providers of primary care). By comparing trends over time for vertically integrating general practices with those that did not merge with another practice or trust and did not change the form of contract they hold, we have found that vertical integration is associated with modest step-change reductions in patients’ rates of the following: A&E attendances (–2%); outpatient attendances (–1%); emergency admissions (–3%); and emergency readmissions (–5%). We found no association with changes in rates of overall inpatient admissions or admissions for ambulatory care sensitive conditions; nor with length of stay. The falls in A&E and outpatient attendance rates are temporary; these rates resume growing at faster rates than for control practices, which offsets the initial step-change reduction within 1 or 2 years. There was little difference in the impact of vertical integration when comparing integration with acute hospital trusts and integration with community trusts.

### Strengths and limitations

We have adopted a pragmatic but robust analytical approach: excluding comparators that are confounded by merger or contract change; and limiting analysis to the pre-February 2020 period to avoid the major impact on all healthcare utilisation that was the consequence of the COVID-19 pandemic. We have restricted analysis to the instances of vertical integration that are supported by reliable hospital activity data for 2 years before the date of vertical integration and 2 years afterwards, in order not to reduce the number of analysable vertical integrations to too small a number. Within these criteria, all eligible practices with acceptable quality data were included. However, it is likely that the full impact of such a major change as a trust taking over the running of a general practice would take longer than 2 years to emerge. The modest sample size of vertically integrated practices is an unavoidable limitation owing to the small (although growing) numbers of general practices that have vertically integrated.

Earlier work by authors of the current study found that some of the general practices that vertically integrated might well have closed otherwise.^6,7^ We have not been able to reconstruct what the impact of this would have been on use of hospital services had practices closed. Instead we have compared hospital utilisation by patients of vertically integrating practices with that of patients of stable practices (that neither vertically integrated, nor merged with another practice, nor changed contract type). Analysis reported elsewhere, shows that general practices that vertically integrate are probably not typical of practices overall.^5^ Hence, although our results matter for places where vertical integration has been implemented as a result of local circumstances and on the basis of local initiative, they should not be extrapolated; for example, to the hypothetical case of vertical integration being undertaken across the NHS in England.

### Comparison with existing literature

The study reported here followed earlier qualitative analysis by some of the same authors at two vertical integration locations in England and one in Wales.^6,7^ A quantitative evaluation of new models of care integration at two locations by Stokes and colleagues,^23^ includes one location that part way through the period studied also introduced vertical integration but analyses changes starting at an earlier date and does not separately identify the impact of vertical integration.

Our findings are broadly consistent with those of the one previous statistical analysis of vertical integration in the literature, which is for a single locality — Wolverhampton (an urban area in the West Midlands of England) — and which took a different approach to identifying a counterfactual.^16^ Looking at the outcome measures included in that study, we similarly found modest reductions in inpatient admissions and emergency readmissions when practices vertically integrate with a trust. We additionally found an impact on A&E attendances that was not found by the Wolverhampton analysis.

### Implications for practice and research

Our analysis has shown that vertical integration in some locations can lead to modest reductions in use of hospital services, which can be assumed to have corresponding, modest, implications for financial savings. But our analysis does not imply a case for vertical integration to be rolled out nationally. It is likely that our data-driven limitation of investigating just 2 years of (monthly) data following vertical integration is too short a period for the full impacts to be revealed. Further research when more time has elapsed would be valuable. We have published separately an analysis of the impact of vertical integration on patient experience and continuity.^19^ Further research into the impact of vertical integration on service coordination would additionally be valuable.

## References

[1] NHS England (2014). Five year forward view.

[2] NHS England (2016). General practice forward view (GPFV).

[3] NHS England (2019). The NHS long term plan.

[4] NHS England (2022). Next steps for integrating primary care: Fuller stocktake report.

[5] Davies C, Saunders CL, Olumogba F (2024). Identifying where hospital and community trusts are managing general practices in England: a service mapping study. BJGP Open.

[6] Sidhu M, Pollard J, Sussex J (2022). Vertical integration of primary care practices with acute hospitals in England and Wales: Why, how and so what? Findings from a qualitative, rapid evaluation. BMJ Open.

[7] Sidhu M, Pollard J, Sussex J (2022). Vertical integration of GP practices with acute hospitals in England and Wales: rapid evaluation. Health Soc Care Deliv Res.

[8] Baker R, Bankart MJ, Rashid A (2011). Characteristics of general practices associated with emergency-department attendance rates: a cross-sectional study. BMJ Qual Saf.

[9] Barker I, Steventon A, Deeny SR (2017). Association between continuity of care in general practice and hospital admissions for ambulatory care sensitive conditions: cross sectional study of routinely collected, person level data. BMJ.

[10] Chauhan M, Bankart MJ, Labeit A, Baker R (2012). Characteristics of general practices associated with numbers of elective admissions. J Public Health (Oxf).

[11] Bankart MJG, Baker R, Rashid A (2011). Characteristics of general practices associated with emergency admission rates to hospital: a cross-sectional study. Emerg Med J.

[12] House of Commons Health and Social Care Committee The future of general practice: fourth report of session 2022–23.

[13] Blom AB, von Bülow LL (2013). Vertical integration across hospital acute care and on-call general practitioners. An evaluation of a cross sectional cooperation model at Odense University Hospital, Southern Region of Denmark. Int J Integr Care.

[14] Comendeiro-Maaløe M, Ridao-López M, Gorgemans S, Bernal-Delgado E (2019). A comparative performance analysis of a renowned public private partnership for health care provision in Spain between 2003 and 2015. Health Policy.

[15] Schwartz PM, Kelly C, Cheadle A (2018). The Kaiser Permanente Community Health Initiative: a decade of implementing and evaluating community change. Am J Prev Med.

[16] Yu V, Wyatt S, Woodall M (2020). Hospital admissions after vertical integration of general practices with an acute hospital: a retrospective synthetic matched controlled database study. Br J Gen Pract.

[17] Iacobucci G (2022). Government wants more GPs to be employed by hospital trusts, says news report. BMJ.

[18] Limb M (2022). What’s behind the government’s plan for hospitals to employ more GPs. BMJ.

[19] Sidhu M, Saunders CL, Davies C (2023). Vertical integration of general practices with acute hospitals in England: rapid impact evaluation. Health Soc Care Deliv Res.

[20] NHS Digital Hospital Episode Statistics (HES).

[21] Gov.uk Contracts finder.

[22] NHS Digital GP and GP practice related data.

[23] Stokes J, Shah V, Goldzahl L (2021). Does prevention-focused integration lead to the triple aim? An evaluation of two new care models in England. J Health Serv Res Policy.

[24] Bardsley M, Blunt I, Davies S, Dixon J (2013). Is secondary preventive care improving? Observational study of 10-year trends in emergency admissions for conditions amenable to ambulatory care. BMJ Open.

